# On the use of resampling tests for evaluating statistical significance of binding-site co-occurrence

**DOI:** 10.1186/1471-2105-11-359

**Published:** 2010-06-30

**Authors:** David S Huen, Steven Russell

**Affiliations:** 1 Department of Genetics, University of Cambridge, Cambridge, CB2 3EH, UK; 2 Cambridge Systems Biology Centre, Cambridge, CB2 1QR, UK

## Abstract

**Background:**

In eukaryotes, most DNA-binding proteins exert their action as members of large effector complexes. The presence of these complexes are revealed in high-throughput genome-wide assays by the co-occurrence of the binding sites of different complex components. Resampling tests are one route by which the statistical significance of apparent co-occurrence can be assessed.

**Results:**

We have investigated two resampling approaches for evaluating the statistical significance of binding-site co-occurrence. The permutation test approach was found to yield overly favourable p-values while the independent resampling approach had the opposite effect and is of little use in practical terms. We have developed a new, pragmatically-devised hybrid approach that, when applied to the experimental results of an Polycomb/Trithorax study, yielded p-values consistent with the findings of that study. We extended our investigations to the FL method developed by Haiminen *et al*, which derives its null distribution from all binding sites within a dataset, and show that the p-value computed for a pair of factors by this method can depend on which other factors are included in that dataset. Both our hybrid method and the FL method appeared to yield plausible estimates of the statistical significance of co-occurrences although our hybrid method was more conservative when applied to the Polycomb/Trithorax dataset.

A high-performance parallelized implementation of the hybrid method is available.

**Conclusions:**

We propose a new resampling-based co-occurrence significance test and demonstrate that it performs as well as or better than existing methods on a large experimentally-derived dataset. We believe it can be usefully applied to data from high-throughput genome-wide techniques such as ChIP-chip or DamID. The Cooccur package, which implements our approach, accompanies this paper.

## Background

A large number of proteins are known to bind DNA in a location-specific manner. These include transcription factors, replication factors and chromatin components. It is widely accepted that individual proteins do not usually act in isolation but form multi-protein effector complexes on DNA. When the binding sites of the individual proteins within a complex are determined by genome-wide high-throughput assays, these complexes are revealed as regions where the binding sites of multiple proteins are clustered. When evaluating the apparent co-localisation of the binding sites of a pair of proteins it is necessary to determine whether they genuinely co-localise or whether the observations could have arisen by chance. Many methods have been proposed for assessing the statistical significance of such clusters (reviewed in [1]). The classical statistical methods rely on obtaining the null distribution for a *statistic *that correlates with the phenomenon of interest. The two key design decisions are therefore the choice of how the statistic is computed and how the null distribution is obtained. We will discuss how we have addressed these questions when considering the merit of a particular test and when devising an improved test.

### Choice of statistic

#### General considerations

Some latitude exists in the choice of statistic for a co-occurrence test. With co-occurrence, we are primarily interested in different DNA-binding factors being located closely together more frequently than might be expected 'by chance', the latter being determined by the null distribution. A useful statistic is expected to be increased by the proximity of the factors *and *the number of clusters of factors in the genome. Using the term *heterogeneous overlap *to refer to overlap between the binding sites of two different factors, one can readily envisage as useful statistics either counting the number of pairs of locations where heterogeneous overlaps occur, or the total number of bases of heterogeneous overlap. With the former, it is the presence or otherwise of an overlap that determines whether to increment the statistic while the latter weights overlaps according to the degree they are overlapped.

We desire a general framework for a statistic that is applicable to different policies for scoring co-occurrence. Additionally, we also wish to divorce the specific scoring policy from the statistical test itself. A co-occurrence matrix can satisfy both objectives.

#### Co-occurrence matrix

We will use the term *binding profile *to refer to the list of locations bound directly or otherwise by a specific DNA-associated factor (DAF). Let  and  be the binding profiles of a pair of DAFs, A and B, respectively and define their joint set of locations as .

**Figure 1:**
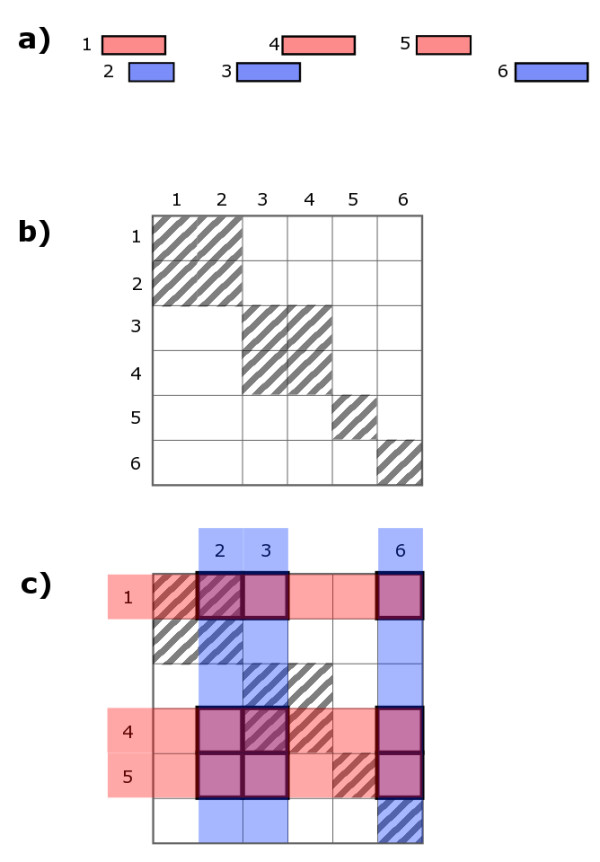
**An example of a co-occurrence matrix**. (a) A binding profile for two proteins, A and B, are displayed schematically in red and blue respectively. The sequence coordinate is along the horizontal axis and each rectangle represents a location. (b) A schematic of the co-occurrence matrix for the joint profile of (a). Locations are numbered sequentially by start coordinate. Rows and columns are associated with factors A and B respectively. The metric is used is whether locations overlap. Overlapped pairs have non-zero *c*_*ij *_and are indicated by diagonal hatching of the element. For example, we see from the matrix that locations 3 and 4 overlap and when they are bound by factors A and B, *c*_34 _is non-zero and therefore the statistic is increased by its value. (c) The binding profiles of A and B shown in (a) have been indicated on the co-occurrence matrix with faint red and blue backgrounds respectively. The elements that will be summed in evaluating the statistic are outlined in bold. These represent every pairwise combination of the two profiles.

**Figure 2:**
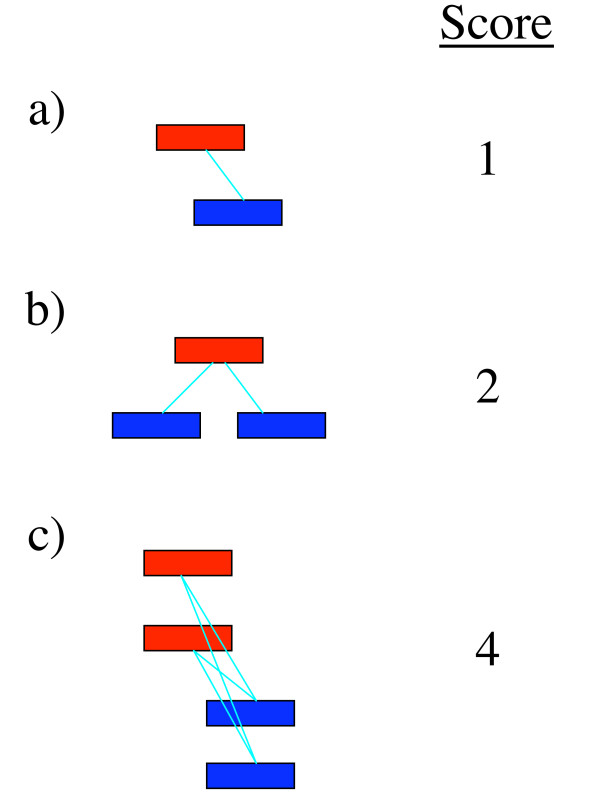
**Statistic used for assessing co-occurrence**. For the purposes of this paper, a simple statistic was used that counted each instance of an overlap between locations bound by different factors. Examples of clusters scoring (a) one, (b) two, and (c) four are shown. Note that in (c), in multiply-assigned locations, each assignment is treated as an independent location.

Further, let there be a co-occurrence matrix, ***C ***with elements, *c*_*ij*_, such that the co-occurrence statistic can be defined as

i.e. the co-occurrence statistic is the sum of the scores of every pairwise combination of locations where one is drawn from those bound by A and the other from one bound by B (Figure 1 with the specific implementation used in this paper described in Figure 2).

The co-occurrence matrix embodies all information relevant to the calculation of the statistic. *c*_*ij *_is the contribution to the statistic when location *i *is bound by A and location *j *is bound by B. That ***C ***is defined in general terms allows its use with a wide range of different approaches to scoring co-occurrence.

For example, if the co-occurrence metric is whether the features overlap irrespective of its extent, *c*_*ij *_is set to unity when an locations *i *and *j *overlap and to zero otherwise. Alternatively, if the number of bases overlapped between two locations is the metric, *c*_*ij *_is set to the number of bases overlapped between locations *i *and *j*. Other scoring strategies can be readily considered and implemented via a co-occurrence matrix in a similar manner. The use of a matrix removes subsequently irrelevant details such as the actual base coordinates and the specific overlap scoring metric from further consideration since it incorporates their entire effect on the calculation of the statistic. Computation of the statistic can therefore be performed during resampling solely by reference to the co-occurrence matrix.

### Choice of null distribution

The difference between statistical methods frequently reduces to the manner in which the null distribution is obtained. With parametric methods, the observed data is used to parameterise one of the classical statistical distributions which is then used as the null distribution. Non-parametric methods use resampling approaches to approximate the null distribution.

Chromatin components naturally bind in a highly non-uniform manner and the null distribution needs to account for this appropriately. Indeed, failure to account for so called bursty sequence effects can result in many false positives [2]. In addition, co-occurrence tests must be cognisant of the provenance of the binding site data. Binding profiles arise from experiments and the mapped sites reflect the actual physical state of chromatin. The contribution of the underlying sequence is therefore already entirely accounted for in the binding profile. In contrast, attempts to identify clustering of sequence-specific proteins by the co-occurrence of their binding site motifs try to reconstruct the chromatin state from sequence: in this case, the binding-site motifs specified by the position-weight matrices (PWMs) and the underlying sequence composition are pertinent to the specification of the null distribution: for example, two similar PWMs will have a large number of co-occurring hits anyway. So while both sources of data generate sets of locations that can be analysed for co-occurrence, the null distributions are likely to be very different. Here, our interest is strictly confined to the simpler case of binding profiles alone and our method is unlikely to be applicable to motif co-occurrence data. A survey of the motif co-occurrence problem can be obtained from papers cited in [3].

Tests based around resampling strategies have been previously explored as a means of accounting for variation in binding site distribution [4]. As they rely on randomising the *observed *binding sites for the factors, they can be expected to automatically account for binding site non-uniformity. In the following sections, we will demonstrate that the utility of these methods is highly dependent on the resampling scheme used.

Resampling strategies rely on sampling a pool of locations to infer the null distribution. The pool can be constituted in two ways. First, it can comprise only the binding profiles of the pair of factors being examined for co-occurrence. Alternatively, it could also be constituted by pooling the binding profiles of a large number of factors, some or all of which are to be investigated for pair-wise co-occurrence. All methods we discuss except for the FL method of Haiminen *et al *use the former approach.

### Resampling-based tests

Resampling methods operate by randomly sampling the observed data to construct a null distribution. When evaluating the statistical significance of co-occurrences between a pair of binding profiles containing *m *and *n *locations respectively, their constituent sites are combined to form a joint pool of locations. Further pairs of binding profiles containing *m *and *n *locations respectively are then repeatedly sampled from this joint pool and scored for co-occurrence to estimate the null distribution of the test statistic.

We can describe resampling formally with two sampling operators ***F***(***X***, *n*) and ***G***(***X***, *n*) that draw *n *elements from ***X***. Then, at each iteration, two new lists of binding sites,  and , the same size as the originals are generated:-

and the distribution of  is used as the null distribution.  and  are the numbers of elements in  and .

In the following sections, we report the results of our investigations into three different resampling strategies, including the previously described permutation test approach.

#### Permutation test

**Figure 3:**
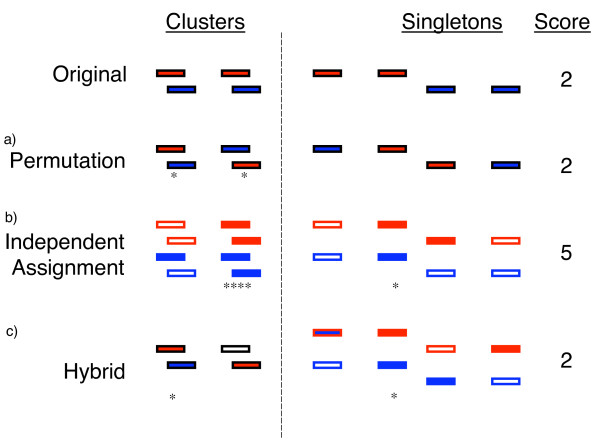
**Examples of permutation schemes**. In the top panel a binding profile in which two factors, A (red) and B (blue), bind at four different locations yielding two overlapped clusters. The colour of outlines indicate how that location can be assigned. A location outlined in black can only be assigned once. Red and blue outlines indicate that location can be assigned to A or B respectively. Assignments are without replacement, i.e. multiple assignments of the same location to the same factor are not permitted. Asterisks show where an overlap is scored. Examples of the combinations that arise from different schemes are shown. (a) Permutation: all locations are assigned to either A or B but not both. No locations are left unassigned. Overlaps can only occur where overlaps have been observed before. (b) Independent assignment. All locations can be assigned to either A or B or both factors. Previously unobserved overlaps arise when the same location is assigned to both factors. (c) Hybrid: Locations previously observed to be involved in co-occurrences can only be assigned to A or B but not both. Singletons can be freely assigned to either factor or both factors.

In this approach, a joint pool of all binding sites is created and  sites randomly assigned to DAF A. The remaining sites are then assigned to DAF B (see Figure 3(a)).

Let ***ρ ***and  be operators for sampling with replacement and without replacement respectively. The permutation test can then be formally described in set notation as:-

#### Independent resampling

Instead of permuting the joint pool of locations, this approach reconstitutes  and  independently (see Figure 3(b)), i.e.

Note that the same location can be assigned to both factors with independent sampling.

#### Hybrid method

The approach here is to split the joint locations into two pools. The first pool comprises locations at which co-occurrence has been observed in the dataset. The second pool comprises the remaining singleton locations.

During resampling, locations in the first pool may either remain unassigned or allocated to one factor only (see Figure 3(c)). Thus, factors co-occur in this pool only when they are assigned to a pair of overlapped locations. Locations in the second pool are freely assigned to the factors. But since the locations in this pool are singletons, overlaps arise only when both factors are assigned to the same location.

A formal statement of this strategy proceeds along the following lines. The locations in  are partitioned into sets of heterogeneously overlapped and singleton locations. We can define the set of singleton locations with reference to the co-occurrence matrix by

and the set of heterogeneously overlapped locations is then

The resampling is then performed by:-

#### Multiple factor methods

Haiminen *et al *proposed and analysed the performance of several interesting approaches that can be applied where the binding sites of many factors are simultaneously known on the same sequence extents, a situation that arises frequently with high-throughput datasets [2].

Two approaches of theirs are of particular interest. In the FL approach, the joint pool comprises all binding sites for all factors in the dataset. Permutation is performed by assigning  sites to DAF A from this pool. A further  sites are then assigned to DAF B from the remaining unassigned members of the pool. The null distribution is the distribution of co-occurrence scores obtained by this permutation method. In the FL(r) method, the binding sites of the first factor, DAF A, remain unchanged while those of the second factor are permuted as described above.

The FL but not the FL(r) approach will be included in our analysis. We are not entirely at ease with treating one of a pair of factors differently from the other nor with the possibility that a co-occurrence may be significant when one factor is fixed but not when the other is fixed.

### Desired behaviour of a co-occurrence significance test

It is important to form some expectation of the p-values that could arise from a sensible method for assessing factor co-occurrence. First, greater significance would be expected when a larger proportion of sites co-occur. For example, one might expect 50 co-occurrences where each factor only bound to 100 sites to yield a lower p-value than if each factor bound to 10000 sites. In addition, given the same proportion of co-occurring sites, a greater significance is expected if the total number of sites is larger.

## Results and Discussion

### A simple model system

A particularly simple co-occurrence model is one with *m *paired co-occurrences out of a total of (*n*_*A *_= ) and (*n*_*B *_= ) binding sites for DAFs A and B respectively.

The behaviour of different approaches to implementing permutation tests will initially be investigated on this analytically tractable system. Unless otherwise stated, the statistic used is the number of co-occurrences as described above and a co-occurrence is deemed to occur if two locations bound by different factors overlapped each other. As we note above, the specific overlap definition used has little influence on the validity or otherwise of the approaches.

### Permutation test

This approach appears to be similar to that reported by Hannenhalli and Levy [4].

#### Permutation test: synthetic data

When a simulation was conducted with *m *= 10 and *n*_*A *_= *n*_*B *_= 10, i.e. wherein all 10 sites bound by A and B were paired, 139 co-occurrences were observed in 100000 resamplings yielding a p-value of 0.00139. When the total number of sites bound was increased to *n*_*A *_= *n*_*B *_= 1000 without change to the number of co-occurrences, 98 co-occurrences were observed which yields an estimated p-value of 0.00098. An apparent anomaly arises therefore where the p-value is only modestly changed when the proportion of co-occurring sites changes from 1 in 2 to 1 in 100. More surprisingly, the p-value actually falls with this change. What is the source of this anomaly? A better understanding can be gained from examining the mathematics behind this approach.

The p-value for the simple model can be derived analytically. The observed number of co-occurrences in this case is also the highest value it could attain - no permutation can cause a co-occurrence at any of the singleton locations since each of these can only be assigned to one of the factors. The p-value is therefore the probability of exactly *m *paired co-occurrences given the values of *n*_*A *_and *n*_*B*_. This probability is, in turn, the product of the probability of having exactly *m *sites of each factor assigned to the overlapped pairs, *P*_*m *_and the probability of them being arranged as pairs, *P*_*pair*_. The former is described by the hypergeometric distribution

The latter is the product of selecting *m *pairs starting with *m *of each factor. The probability of each pair is described by the hypergeometric distribution from the number of sites left to assign. On simplifying

For any given ratio of *n*_*A *_to *n*_*B*_, *p*_*m *_changes little with increasing *n *= *n*_*A *_+ *n*_*B*_. For example, with *n*_*A *_= *n*_*B *_= 20, the probability of assigning 10 sites to each factor is 0.247. With *n*_*A*_= *n*_*B *_= 1000, it is 0.177. The p-value is therefore dominated by *P*_*pair*_, i.e. it is the number of overlaps that determines the p-value: the proportion of sites overlapped has little influence on it. For example, *P*_*pair *_for 10 overlaps is 0.0055 which is the upper limit for p-value irrespective of the total number of sites. With 20 overlaps, *P*_*pair *_= 7.6 × 10^-6 ^which guarantees statistical significance on almost any reasonable threshold irrespective of how small a proportion of the total number of sites bound that might be. This does not accord with what might be deemed reasonable behaviour from a co-occurrence test.

#### Permutation test: other implications

As previously noted, the permutation test approach appears to have been used by Hannenhalli and Levy [4] albeit in a different statistical framework. We were interested to determine whether their framework circumvents the drawbacks we identified with a permutation test approach assessed with the hypergeometric distribution.

**Figure 4:**
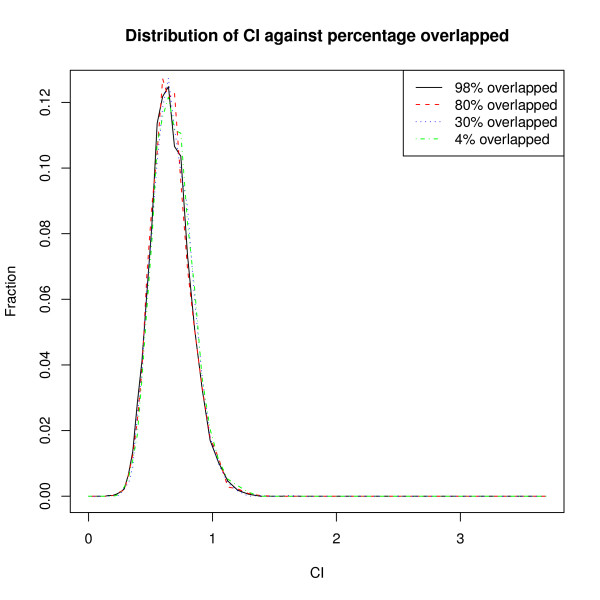
**CI distribution for different degrees of overlap**. The distribution of co-occurrence index was computed with 5000 resampling iterations for 40 overlapped pairs and varying numbers of singletons (1/10/100/1000 singletons per PWM).

Hannenhalli and Levy studied the co-occurrence of hits generated by a pair of position-weight matrices (PWMs) and counted the number of times hits from different PWMs were within some threshold distance of each other. They then computed a co-localization index (CI):-

where *N*_*ij *_is the number of co-occurrences observed between two PWMs, *i *and *j*, and *R*_*ij*_is the number observed after a permutation. Repeated permutations of the dataset can be expected to yield differing values of *R*_*ij *_and it is not clear how the value of *R*_*ij *_used above is selected. Further, it should also be noted that both the log-ratio and the ratio have been used to define *CI *[4,5]. To get an idea as to how the distribution of *CI *varies with the fraction of sites overlapped, we simulated 5000 resamplings of a simple model having 40 paired overlaps and a varying number of singleton sites (Figure 4). It is clear that the distribution of *CI *varies little with the fraction of co-occurring sites and is almost wholly determined by the absolute number of overlapped sites. Hannenhalli and Levy [4] commented on the high frequency of co-occurring transcription factor binding and the paradox that higher-order co-occurrences were not enriched amongst factors showing highly-significant pairwise co-occurrences. While co-occurring PWM pairs will be expected to yield many overlapped pairs and therefore generate high *CI *scores, it could be that some of the highly scoring PWM pairs had large number of hits such that a small fraction of overlaps is suficient to yield enough overlaps to give a high *CI *thereby causing those factors to be incorrectly identified as co-occurring pairs.

### Independent resampling

A major reason for the highly significant p-values observed with the permutation test approach is that the observed score is maximal and there are so many ways in which *m *sites of each factor can be arranged to result in fewer overlaps. Instead of permuting the available sites, if two independent draws of size *n*_*A *_and *n*_*B *_are made, it is possible for the same location to be assigned to both factors (see Figure 3(b)). The observed score is then no longer maximal since overlaps can potentially occur at any location. Further, when both locations involved with the observed overlaps are assigned to both factors, four overlaps can be scored instead of the single overlap observed (e.g. fig 3(b)). This change in sampling procedure has the effect of reducing statistical significance since the observed score can be matched or exceeded in many different ways.

In contrast to the permutation test approach, which leads to overly significant p-values, statistical significance was never achieved with independent resampling. For example, using synthetic data with 20 locations, all presenting as overlapped pairs, a surprisingly high value of 0.64 is obtained. This arises because whenever a pair of overlapped locations has each of its locations assigned to both factors, it increases the score by four. This is four times that obtained when two isolated locations, each bound by a different factor, overlap each other.

### Hybrid approach

The behaviour of this model is expected to be intermediate between permutation test and independent draws in that the first pool is treated with the former strategy while the second is treated with the latter.

#### Hybrid approach: synthetic data

With a profile consisting of paired overlaps only, as with the earlier example with 10 pairs (*m *= 10), the result is identical to that obtained from the permutation test strategy, i.e. the p-value is approximately 0.005. However, merely adding 5 singletons of each factor to the model raises the p-value to approximately 0.12. We now compare other scenarios against this baseline example. With more pairs, the p-value becomes more tolerant of singletons. For example, with *m *= 20, adding 5 singletons to each factor yields a p-value of around 0.001 (as opposed to 0.12 with the baseline). We expect that for the same proportion of overlapped locations, the p-value should fall with an increase in the total number of locations. Adding ten locations to each factor with *m *= 20 gives the same proportion of overlapped locations as the baseline but yields a p-value of around 0.02 (which is lower than the baseline value of 0.12). Both these behaviours are concordant with the desired behaviour of an appropriate co-occurrence test.

The asymptotic behaviour of a statistic *S*_*hyb *_computed by the hybrid method can be considered for the case where a binary scoring is used. At one extreme, when there are no singleton sites, the method is entirely equivalent to the permutation test and should yield significant p-values provided the number of sites overlapped is large enough. At the other extreme, when a negligible fraction of the locations are overlapped, the distribution of *S*_*hyb *_can be expected to approach that where *n*_*A *_As and *n*_*B *_Bs bind a total of *n*_*A *_+ *n*_*B *_sites. The probability mass function (pmf) of *S*_*hyb *_for this limiting case is

where 0 ≤ *k *≤ min(*n*_*A*_, *n*_*B*_). (derivation in Materials and Methods section).

As the average value of *S*_*hyb *_is then *n*_*A*_*n*_*B*_*/*(*n*_*A *_+ *n*_*B*_), we may expect that statistical significance will only be achieved if the observed number of overlaps considerably exceeds this.

#### Hybrid approach: Polycomb/Trithorax data

The hybrid technique was tested on data published by Schuettengruber *et al *[6]. The authors published ChIP-chip [7] binding profiles for Polycomb and Trithorax proteins (PC, TRX-N, TRX-C), other chromatin proteins, Polyhomeotic, Pleiohomeotic, Pleiohomeotic-like, Dorsal switch protein-1, GAGA factor (PH, PHO, PHOL, DSP1, GAF) as well as histone marks (Me3K4, Me3K27) in *Drosophila melanogaster*. In all, ten chromatin components bound over 28068 locations were examined.

In this test, the presence of co-occurrence was investigated for all possible pairs of binding profiles. Of the 45 potential pairs, 11 highly-significant co-occurring pairs (p-value < 0.05) were identified. These were PC-Me3K27, PC-PH, Me3K27-PH, Me3K4-PHO, Me3K4-PHOL, PHO-PHOL, Me3K4-TRX-N, DSP1-GAF, DSP1-PHO, PHOL-TRX-N and PHO-TRX-N.

**Figure 5:**
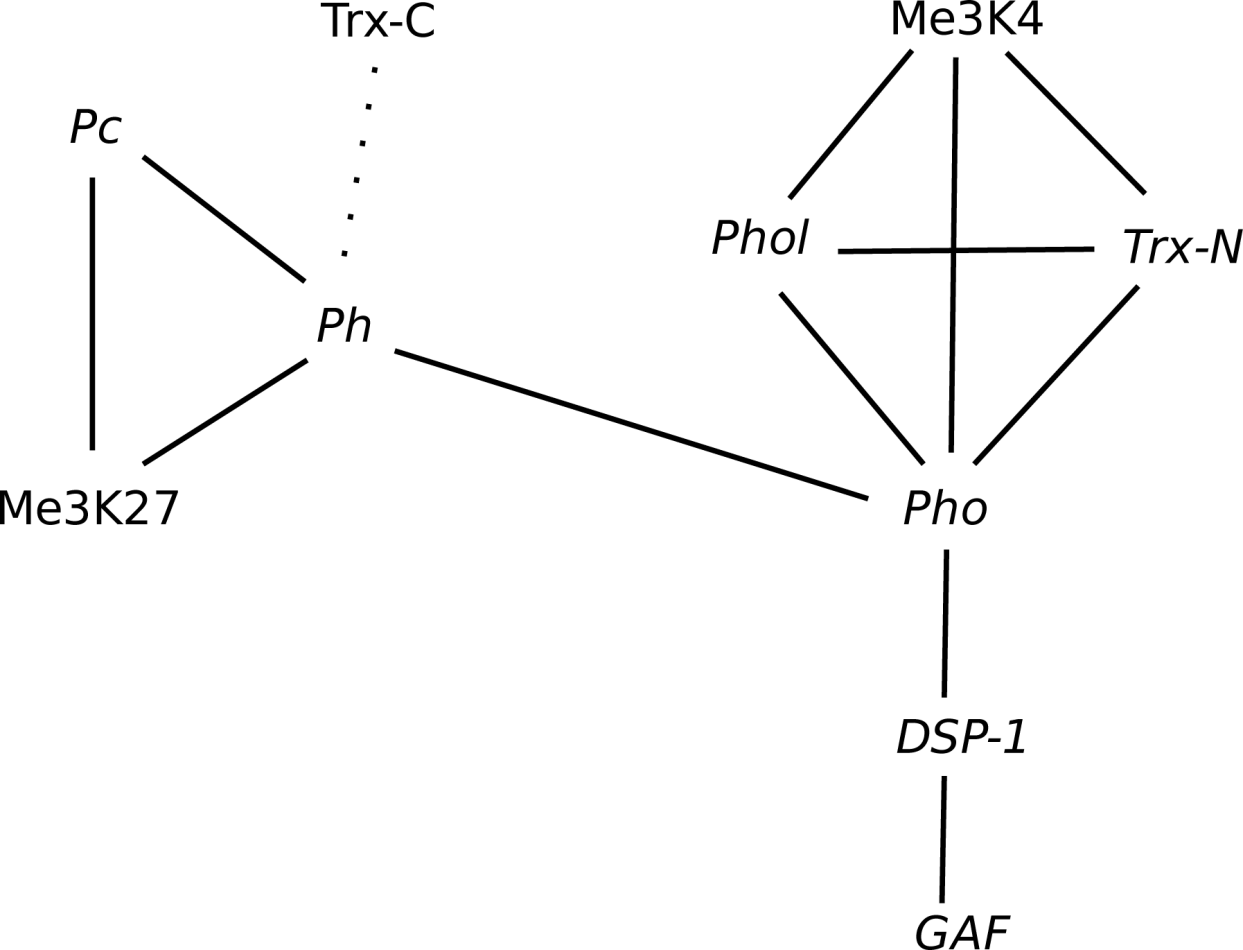
**Clusters of statistically significant co-occurring pairs in Polycomb/Trithorax dataset**. This figure shows two clusters formed by statistically significant pairwise co-occurrences (p-value << 0.001; marked by bold lines) between binding profiles of factors analysed in Schuettengruber *et al *[6]. Two lower confidence co-occurrences are shown with dashed (p-value < 0.05) and dotted lines (p-value < 0.1) respectively.

The co-occurrences divided the factors nicely into two clusters (Figure 5) with a single co-occurrence linking the clusters (PH-PHO). The first cluster includes the two Polycomb recruitment complex I (PRC1) proteins (PH, PC) while the Pleiohomeotic recruitment complex (PhoRC) proteins (PHO, PHOL) localise with the other cluster. It was particularly pleasing to detect a PH-PHO co-occurrence since these are similar proteins that bind the same target sequence *in vitro *but have somewhat different binding profiles *in vivo *[6]. Unlike PHOL, which only interacts with the PRC2 component Esc [8], PHO is known to interact directly with the PRC1 components PC and PH as well as with Esc [9].

Trithorax is known to be cleaved into two independently acting fragments, TRX-N and TRX-C [10,11]. A very weak score for the PH-TRX-C co-occurrence was again consistent with the finding that while TRX-N is associated with Me3K4-marked sites, TRX-C is associated with PRC1 complexes [6].

**Figure 6:**
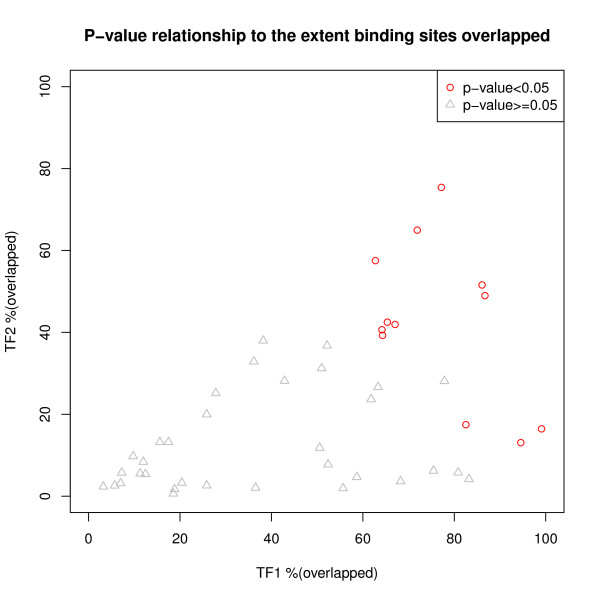
**Relationship between p-value and degree of overlap**. The data in this plot is derived from the Polycomb/Trithorax dataset [6]. The axes represent the percentage of binding sites overlapped for the a pair of factors, arranged such that the larger percentage is plotted on the *x*-axis. Factors with significant co-occurrence are plotted in red while those without are plotted in black.

We were interested in how the p-value behaved with different degrees of overlap between factors and in particular where the threshold between statistically significant and insignificant overlap lay (Figure 6). It is noteworthy that this method does not only detect cases where both sets overlap extensively, i.e. where percentage overlaps in both cases exceed 50%, but also detects occasions where one factor almost wholly co-occurs with a subset of binding sites of the other factor. Our hybrid method appears to detect co-occurrences broadly consistent with known interactions. A summary of results is shown in Table 1 with the p-values obtained by each of the methods described in this paper. Note that our earlier objections to the permutation test and the independent resampling approach have been amply confirmed when applied to this dataset.

**Table 1:** Summary table of Polycomb/Trithorax analysis

Factors		#(overlapped)/#total (percentage)		P-value			
				**Perm**	**Indep**	**Hai**	**Hyb**
**TF1**	**TF2**	**TF1**	**TF2**	**Test**	**Res**	**FL**	**Mtd**

**Dsp1**	**GAF**	**1329/1982 (67%)**	**1266/3019 (41.9%)**	<**0.001**	~1	<**2e-04**	<**2e-04**
Dsp1	Me3K27	193/1982 (9.74%)	242/2480 (9.76%)	<0.001	~1	~1	~1
Dsp1	Me3K4	1225/1982 (61.8%)	1158/4893 (23.7%)	<0.001	~1	< 2e-04	~1
Dsp1	Pc	309/1982 (15.6%)	279/2110 (13.2%)	<0.001	~1	~1	~1
Dsp1	Ph	234/1982 (11.8%)	223/441 (50.6%)	<0.001	~1	< 2e-04	~1
**Dsp1**	**Pho**	**1272/1982 (64.2%)**	**1281/3152 (40.6%)**	<**0.001**	~**1**	<**2e-04**	<**2e-04**
Dsp1	Phol	1034/1982 (52.2%)	1085/2951 (36.8%)	<0.001	~1	< 2e-04	~1
Dsp1	TrxC	123/1982 (6.2%)	126/167 (75.4%)	<0.001	~1	< 2e-04	~1
Dsp1	TrxN	1255/1982 (63.3%)	1297/4868 (26.6%)	<0.001	~1	< 2e-04	~1
GAF	Me3K27	253/3019 (8.38%)	297/2480 (12.0%)	<0.001	~1	~1	~1
GAF	Me3K4	1539/3019 (51%)	1528/4893 (31.2%)	<0.001	~1	0.11	~1
GAF	Pc	399/3019 (13.2%)	369/2110 (17.5%)	<0.001	~1	~1	~1
GAF	Ph	234/3019 (7.75%)	231/441 (52.4%)	<0.001	~1	< 2e-04	~1
GAF	Pho	1147/3019 (38%)	1204/3152 (38.2%)	<0.001	~1	< 2e-04	~1
GAF	Phol	993/3019 (32.9%)	1067/2951 (36.2%)	<0.001	~1	< 2e-04	~1
GAF	TrxC	110/3019 (3.64%)	114/167 (68.3%)	<0.001	~1	< 2e-04	~1
GAF	TrxN	1294/3019 (42.9%)	1371/4868 (28.2%)	<0.001	~1	~1	~1
Me3K27	Me3K4	174/2480 (7.02%)	155/4893 (3.17%)	<0.001	~1	~1	~1
**Me3K27**	**Pc**	**1914/2480 (77.2%)**	**1591/2110 (75.4%)**	<**0.001**	~**0.73**	<**2e-04**	<**2e-04**
**Me3K27**	**Ph**	**433/2480 (17.5%)**	**364/441 (82.5%)**	<**0.001**	~**0.76**	<**2e-04**	<**2e-04**
Me3K27	Pho	641/2480 (25.8%)	629/3152 (20.0%)	<0.001	~1	0.91	~1
Me3K27	Phol	80/2480 (3.23%)	69/2951 (2.34%)	<0.001	~1	~1	~1
Me3K27	TrxC	116/2480 (4.68%)	98/167 (58.7%)	<0.001	~1	< 2e-04	~1
Me3K27	TrxN	141/2480 (5.69%)	126/4868 (2.59%)	<0.001	~1	~1	~1
Me3K4	Pc	264/4893 (5.4%)	263/2110 (12.5%)	<0.001	~1	~1	~1
Me3K4	Ph	84/4893 (1.72%)	83/441 (18.8%)	<0.001	~1	~1	~1
**Me3K4**	**Pho**	**1921/4893 (39.3%)**	**2027/3152 (64.3%)**	<**0.001**	~**1**	<**2e-04**	<**2e-04**
**Me3K4**	**Phol**	**2396/4893 (49%)**	**2559/2951 (86.7%)**	<**0.001**	~**1**	<**2e-04**	<**2e-04**
Me3K4	TrxC	31/4893 (0.634%)	31/167 (18.6%)	<0.001	~1	~1	~1
**Me3K4**	**TrxN**	**3179/4893 (65%)**	**3500/4868 (71.9%)**	<**0.001**	~**1**	<**2e-04**	<**2e-04**
**Pc**	**Ph**	**347/2110 (16.4%)**	**437/441 (99%)**	<**0.001**	~**1**	<**2e-04**	<**2e-04**
Pc	Pho	587/2110 (27.8%)	793/3152 (25.2%)	<0.001	~1	2e-04	~1
Pc	Phol	153/2110 (7.25%)	169/2951 (5.73%)	<0.001	~1	~1	~1
Pc	TrxC	122/2110 (5.78%)	135/167 (80.8%)	<0.001	~1	< 2e-04	~1
Pc	TrxN	238/2110 (11.3%)	269/4868 (5.53%)	<0.001	~1	~1	~1
**Ph**	**Pho**	**417/441 (94.6%)**	**412/3152 (13.1%)**	<**0.001**	~**1**	<**2e-04**	<**2e-04**
Ph	Phol	90/441 (20.4%)	97/2951 (3.29%)	<0.001	~1	~1	~1
*Ph*	*TrxC*	*124/441 (28.1%)*	*130/167 (77.8%)*	*<0.001*	~1	*< 2e-04*	*~0.059*
Ph	TrxN	114/441 (25.9%)	127/4868 (2.61%)	<0.001	~1	~1	~1
**Pho**	**Phol**	**1813/3152 (57.5%)**	**1852/2951 (62.8%)**	<**0.001**	~**1**	<**2e-04**	<**2e-04**
Pho	TrxC	131/3152 (4.16%)	139/167 (83.2%)	<0.001	~1	< 2e-04	~1
**Pho**	**TrxN**	**2060/3152 (65.4%)**	**2069/4868 (42.5%)**	<**0.001**	~**1**	<**2e-04**	<**2e-04**
Phol	TrxC	61/2951 (2.07%)	61/167 (36.5%)	<0.001	~1	0.15	~1
**Phol**	**TrxN**	**2540/2951 (86%)**	**2510/4868 (51.6%)**	<**0.001**	~**1**	<**2e-04**	<**2e-04**
TrxC	TrxN	93/167 (55.7%)	94/4868 (1.93%)	<0.001	~1	0.25	~1

Pilot runs of 100 resamplings were performed to filter out clearly non-co-occurring pairs. Thereafter, 5000 resamplings were used for candidate pairs when testing the hybrid method. Tests using the permutation test approach were restricted to 1000 resamplings. Pairs with significant p-values by the hybrid method are indicated in **bold**(p-value ≤ 0.05) and *italics*(p-value ≤ 0.1).

### FL method

An experimental implementation of the FL method [2] was used for the following studies.

#### FL method: synthetic data

Where the dataset consists of only the pair of factors of interest the FL method is identical to the permutation test. However, this is not the context for which the FL method is intended and we examine the effect of additional binding site data from further factors. Consider two factors present as 40 co-occurring pairs. The p-value obtained over 100000 trials is, as expected from our foregoing permutation test analysis, very low: < 1 × 10^-5^. The presence of other factors with a very similar pattern of binding can be examined by adding further pairs of factors with the same binding sites as the first pair. The p-value escalates rapidly on doing so, to 0.012, 0.108 and 0.198 on adding one, two and three replicates respectively. If the effect of the presence of a factor with a disjoint set of binding sites can be readily seen by adding to the four-replicate set, a further factor binding to 100 novel non-overlapping sites. The p-value then declines precipitiously from 0.198 to very significant score of ≈ 1 × 10^-4^. The synthetic data analysis presented above illustrates that the FL method is potentially sensitive to the choice of factors in the dataset, which can be expected given that the null distribution is obtained from combining all binding sites in the dataset. Where factors co-occur frequently, the statistical significance of an observed co-occurrence is reduced accordingly. Correspondingly, where that is not the case, an observed co-occurrence is more readily deemed statistically significant.

#### FL method: Polycomb/trithorax data

To further investigate the potential of the FL method, it was applied to the same Polycomb/Trithorax data previously examined with the hybrid method. A summary of the results from this method are juxtaposed with the results of all other methods in Table 1. In general, with this dataset, the FL method reported more significantly co-occurring pairs than our own hybrid method (27 vs 11 of the 45 possible pairs). All pairs scored as co-occurring by the hybrid method were also co-occurring by the FL method indicating that the FL method was either more sensitive/less conservative than the hybrid method.

Given our concerns over the sensitivity of the FL method to the choice of factors in a dataset, we also determined the effect of removing each factor in turn from the dataset and repeating the analysis. In general, the FL method was found to be quite robust to these exclusions. The results remained unchanged when any one of five factors were excluded (GAF, Me3K4, Me3K27, Pc, TrxN). Exclusion of one of Dsp1, Ph, Pho, Phol or TrxC caused the GAF-Me3K4 co-occurrence to become statistically significant. In addition excluding Pho also caused the Phol-TrxC co-occurrence to be deemed statistically significant. The Suppressor of Hairy-wing insulator protein, Su(Hw), binds at 3794 sites in the *Drosophila *genome, almost all of which are distinct from those of other known chromatin components and its addition to this dataset may be expected to raise the statistical significance of co-occurrences between other factors. This was indeed observed, with both the GAF-Me3K4 and Phol-TrxC co-occurrences already mentioned above becoming significant, as well as three previously non-significant co-occurrences, GAF-TrxN, Me3K27-Pho and TrxC-TrxN. It is noteworthy that while sites in the Su(Hw) binding profile only comprise around 15% of the total original number of locations, they were enough to drive a further five candidate co-occurring pairs to significance.

#### Performance

As with other resampling methods, our hybrid method test is computationally expensive, especially when low p-value thresholds are required. The current implementation is written in pure R and is single-threaded. The user time for analysing the PC-Me3K27 co-occurrence data with 5000 resamplings was 1231 s on a Core 2 Quad Xeon T5600 (1.83 GHz). To improve compute times we exploited the inherent parallelism in the resampling process, utilising the R interface to a Message-Passing interface (MPI) implementation, Rmpi [12,13]. When run on a dual Core 2 Quad Xeon T5600 with six slave processes, the elapsed time for the PC-Me3K27 task fell to 328 s, a reduction of 3.75-fold which amounts to an acceptable 0.8-fold per slave process. Further speedup may be possible by reimplementing parts of the code in a lower-level language.

## Discussion

Our analysis of four different approaches to the use of permutation tests showed the p-values obtained to be highly sensitive to the manner in which the resampling is done. Two aspects are of concern. First, the independent sampling approach cannot be useful in any practical context as it is incapable of yielding significant p-values under any practical conditions. It readily explains why it has not been previously encountered. Of greater concern is the permutation test approach. This method readily yields highly significant p-values with our statistical framework even in cases that should not warrant it. The permutation test approach has been previously used for co-occurrence analysis and we urge caution be used when interpreting data obtained in this way. The FL and hybrid methods were found to be effective on a real-world dataset with the latter being more conservative on this dataset: all pairs scored as significantly co-occurring with the latter were similarly scored with the former.

It is difficult to determine which method is more appropriate. Clustering of factors represent a multi-way co-occurrence which may not be adequately detected/rejected by the presence or otherwise of a pairwise co-occurrence. The use of the FL method is sensitive to the selection of factors used to determine the null distribution and some thought should go into selecting appropriate factors to include in the dataset. In particular, a data mining exercise screening large numbers of binding profiles for co-occurrences might favour the inclusion of the hybrid method in the test repertoire because of its insensitivity to factor selection. Given the limited data available to validate the relative performance of the methods with regard to selectivity and sensitivity, it may be advantageous to use both. Generally, resampling tests are limited in the range of p-values they can yield by the number of resamplings performed and the use of two methods with different sensitivities may allow the very high confidence co-occurrences to be differentiated from the weaker ones. To conclude, we report the development of a hybrid approach based on pragmatic grounds that we believe has utility. Our evaluation, using a comprehensive real-world data set, indicates that the derived co-occurrence data appear reasonable. Indeed, we believe our approach is conservative in that it assumes that all singletons are capable of generating a co-occurrence. While the method may be computationally expensive, it is no more so than other techniques relying on resampling.

An initial release of the Cooccur package implementing our method accompanies this paper (Additional file 1).

## Conclusions

We have proposed a hybrid approach to sampling in a permutation test for detecting pairwise co-occurrences of factor binding. We have also demonstrated that the method is applicable to real-world data and yields results consistent with previous expectations. It is likely to be useful for analysis of data from high-throughput genome-wide screens of factor binding.

## Methods

### Software

All statistical manipulations were implemented in R, a dialect of the S language [14].

### Assessment of co-occurrence

The co-occurrence table used in this study was defined with binary weights, with a unit weight used for overlapping locations. Fig. 3(c) shows an example of our implementation works.

### Extension to multiple contigs

The technique is described above with all locations being on the same sequence. However, as this technique is concerned with assessing co-occurrence only, it is possible to build a co-occurrence table across multiple sequences. Note that locations on different sequences can never result in an overlap with each other and the overlap table can therefore be assembled a contig at a time.

To compute the co-occurrence table for the entire dataset, the locations were searched for overlaps, one sequence at a time and overlaps were appended to the table.

### Runtime optimisations

When determining statistical significance of co-occurrence between a pair of factors, a short survey run of 100 samples with a p-value cut-off of 0.1 was used to filter out factors that clearly do not co-occur. A further 5000 iterations was then performed and an estimate of the p-value obtained from that.

### Derivation of non-overlapped site distribution

In this case, we have a total of *h *= *n*_*A *_+ *n*_*B *_sites, all singletons and we wish to determine the probability of obtaining an *k *sites which are assigned to both DAFs A and B where 0 ≤ *k *≤ min(*n*_*A*_, *n*_*B*_).

Then, the probability of a co-occurrence of size *k *is

## List of abbreviations used

ChIP-chip: chromatin immunoprecipitation on chip; ChIP on array; DamID: CI: co-localization index; DNA adenine methyltransferease identification; DAF: DNA-associated factor; PWM: position-weight matrix

## Authors' contributions

DH conceived the approach used in this study, wrote the software used in this study and characterised its performance on published experimental data. SR directs the research group. Both authors wrote the paper.

## Supplementary Material

Additional file 1 **The Cooccur package**. A development version as installation package suitable for installation with the R CMD INSTALL command. Subsequent versions will be released via Bioconductor. Click here for file
