# Supplementary material for: On the use of resampling tests for evaluating statistical significance of binding-site co-occurrence
Source: BMC Bioinformatics. 2010 Jun 30;11:359. doi: 10.1186/1471-2105-11-359 (PMC2910723; doi:10.1186/1471-2105-11-359)
Supplement: Additional file 1 — The Cooccur package. A development version as installation package suitable for installation with the R CMD INSTALL command. Subsequent versions will be released via Bioconductor. [file 1471-2105-11-359-S1.GZ › Cooccur/inst/doc/demo.pdf]

Using the Cooccur package

David S. Huen

August 27, 2009

We will show an example of how to use `Cooccur` together with `Rmpi` to process chromatin profiles. The data is a subset of that produced by [Schuettengruber, 2009] in which different chromatin components were found to partition into different types of clusters.

I assume that some version of `MPI` and `Rmpi` have been successfully installed on the machine. `Cooccur` does not require `MPI`. It executes on a single thread when that is the case but very significant acceleration is possible when `MPI` and `Rmpi` are available. `Rmpi` can be initialised and various cleanup functions instituted with:-

```
> if (!is.loaded("mpi_initialize")) {
+     library("Rmpi")
+ }
> mpi.spawn.Rslaves(nslaves = 6)

        6 slaves are spawned successfully. 0 failed.
master (rank 0, comm 1) of size 7 is running on: zaitoichi
slave1 (rank 1, comm 1) of size 7 is running on: zaitoichi
slave2 (rank 2, comm 1) of size 7 is running on: zaitoichi
slave3 (rank 3, comm 1) of size 7 is running on: zaitoichi
slave4 (rank 4, comm 1) of size 7 is running on: zaitoichi
slave5 (rank 5, comm 1) of size 7 is running on: zaitoichi
slave6 (rank 6, comm 1) of size 7 is running on: zaitoichi
```

A key step is the the invocation of `mpi.spawn.Rslaves`. This call spawns a number of slaves to execute R code and it should spawn the maximum available number of slaves when called without an argument. However, this does not appear to work on all platforms and an explicit request may be necessary in that case. If you should be using a multiprocessor machine, a reasonable number of slaves could be the number of processors available. When the number of slaves exceeds the number of processors, performance is degraded.

```
> .Last <- function() {
+     if (is.loaded("mpi_initialize")) {
+         if (mpi.comm.size(1) > 0) {
+             print("Please use mpi.close.Rslaves() to close slaves.")
+             mpi.close.Rslaves()
+         }
+         print("Please use mpi.quit() to quit R")
+         .Call("mpi_finalize")
```

```
+     }
+ }
> test <- mpi.setup.sprng()
```

When using multi-processor setups, care must be paid to the generation of random numbers. The statistics from the permutation test are supposed to be independent and random numbers used must therefore be independent on each process. The usual random number generator (RNG) can yield very correlated results if invoked on different processors. Two specialist RNGs are available that deal with this, `Rsprng` and `rlecuyer`. We use `Rsprng` in this example.

When wishing to look at the co-occurrence of a whole range of factors, it is useful to use the `explore_pairs` function. The package comes with chromatin profiles for Polycomb (Pc), Polyhomeotic (Ph) and the histone modifications, Me3K4 and Me3K27. We can examine all pairwise co-occurrences with

```
> library(Cooccur)
> data(Pc, Ph, Me3K4, Me3K27)
> explore_pairs(c("Pc", "Ph", "Me3K4", "Me3K27"))
> print_result(Pc_Ph_result)
```

```
Co-occurrence statistic:  452
No. of TF1 sites overlapped:  347  out of  2110  ( 16.4 %)
No. of TF2 sites overlapped:  437  out of  441  ( 99 %)
No. of samples:  1000
P-value <  0.000999
```

`explore_pairs` will run always run a pilot permutation test with a lower number of resamplings (default 100) and check from the p-value that there is at least some possibility of oc-occurrence before committing to a full run. The selection of the number of resamplings for the full run depends on the situation. 1000 resamplings are probably adequate for screening purposes but perhaps something quite extensive might be warranted for key pairs prior to publication.

`explore_pairs` saves results into your base environment by default and names them according to the pair of profiles used in the analysis. For example, the result of the permutation test for co-occurrence of Pc and Ph is `Pc_Ph_result` and that has been displayed with `print_result`.

However, it would be more useful to collate the results of all tests into a summary data frame. This can be done with `dump_pairs_as_frame`:-

```
> results <- dump_pairs_as_frame(c("Pc", "Ph", "Me3K4", "Me3K27"))
> subset(results, TRUE, select = c(tf1, tf2, pvalue))
```

```
     tf1   tf2      pvalue
1 Me3K27 Me3K4 1.000000000
2 Me3K27    Pc 0.000999001
3 Me3K27    Ph 0.000999001
4  Me3K4    Pc 1.000000000
5  Me3K4    Ph 1.000000000
6     Pc    Ph 0.000999001
```

It will be noted from the above that Pc, Ph and Me3K27 co-occur but Me3K4 does not co-occur with the others. This is as expected, the first three but not the last are associated with PRC1 complexes.

On completion, `MPI` should be shut down and cleaned up:-

```
> mpi.close.Rslaves()

[1] 1
```

The code installed in the preamble will clean up if you don't.

References

[Schuettengruber, 2009] Schuettengruber,B., Ganapathi,M., Leblanc,B., Portoso,M., Jasheck,R., Tolhuis,B., van Lohuizen,M., Tanay,A., Cavalli,G. (2009) Functional anatomy of Polycomb and Trithorax chromatin landscapes in *Drosophila* embryos. *Plos Biology*, 7, e1000013..
